# COVID-19 and Heart Failure: From Epidemiology During the Pandemic to Myocardial Injury, Myocarditis, and Heart Failure Sequelae

**DOI:** 10.3389/fcvm.2021.713560

**Published:** 2021-08-10

**Authors:** Leonardo Italia, Daniela Tomasoni, Stefano Bisegna, Edoardo Pancaldi, Lorenzo Stretti, Marianna Adamo, Marco Metra

**Affiliations:** Cardiology, ASST Spedali Civili and Department of Medical and Surgical Specialties, Radiological Sciences and Public Health, University of Brescia, Brescia, Italy

**Keywords:** COVID-19, SARS-CoV-2 infection, heart failure, myocardial injury, epidemiology, myocarditis, pathophysiology

## Abstract

A close and intriguing relationship has been suggested between heart failure (HF) and coronavirus disease 2019 (COVID-19). First, COVID-19 pandemic represented a global public health emergency in the last year and had a catastrophic impact on health systems worldwide. Several studies showed a reduction in HF hospitalizations, ranging from 30 to 66% in different countries and leading to a subsequent increase in HF mortality. Second, pre-existing HF is a risk factor for a more severe clinical course of COVID-19 and an independent predictor of in-hospital mortality. Third, patients hospitalized for COVID-19 may develop both an acute decompensation of chronic HF and *de-novo* HF as a consequence of myocardial injury and cardiovascular (CV) complications. Myocardial injury occurred in at least 10% of unselected COVID-19 cases and up to 41% in critically ill patients or in those with concomitant CV comorbidities. Few cases of COVID-19-related acute myocarditis, presenting with severe reduction in the left ventricular (LV) ejection fraction and peculiar histopathological findings, were described. However, recent data suggested that COVID-19 may be associated with both systolic and diastolic LV dysfunction, with LV diastolic impairment, pulmonary hypertension, and right ventricular dysfunction representing the most frequent findings in echocardiographic studies. An overview of available data and the potential mechanisms behind myocardial injury, possibly leading to HF, will be presented in this review. Beyond the acute phase, HF as a possible long-term consequence of cardiac involvement in COVID-19 patients has been supposed and need to be investigated yet.

## Introduction

Coronavirus disease 2019 (COVID-19) rapidly spread around the world becoming a global public health emergency. It is caused by a novel enveloped, positively stranded RNA beta coronavirus named severe acute respiratory syndrome coronavirus 2 (SARS-CoV-2) (1). So far, more than one hundred million of confirmed COVID-19 cases can be counted worldwide, with a total of more than three million deaths, as of June 1, 2021, according to the World Health Organization (2).

Although COVID-19 was initially considered a respiratory disease, it has rapidly become clear that a multiorgan involvement was common. In particular, the heart often represents a target organ and patients may develop heart failure (HF) (3–6).

Of note, the link between COVID-19 and HF is more complex. First, COVID-19 pandemic has an impact on HF management and a reduction of hospitalizations due to HF has been shown during the pandemic period, possibly leading to an increase in HF mortality. Second, history of HF is a risk factor for a more severe clinical course of COVID-19. Third, HF can be a consequence of COVID-19-related myocardial damage.

The aim of this review is to describe the epidemiology of HF during the pandemic, the role of cardiac injury and HF in COVID-19, and its pathogenetic mechanisms.

## Heart Failure Epidemiology During COVID-19

COVID-19 pandemic upsets the epidemiology and the management of acute HF. Urgent cardiovascular (CV) hospital admission showed a general decline during the pandemic period, with also a delay in urgent care and an increased risk of complications (7, 8). Similarly, several studies reported a reduction in HF hospitalizations ranging from 30 to 66% (Table 1) (9–13). An analysis from a tertiary Heart Failure Unit in London showed that the number of HF hospitalizations had a significant decline by 66% during the COVID-19 pandemic, compared both with a pre-COVID period in the same year and the corresponding time periods from 2017 to 2019 (9). Patients hospitalized for HF during the pandemic were sicker, with higher rates of NYHA class III or IV symptoms and severe peripheral oedema, which are known predictors of poor outcomes in acute HF. The authors speculated that patients with less severe acute HF might have avoided presenting to hospital during the pandemic, due to the fear of acquiring infection (9). Further studies aimed to compare not only the rates of HF hospitalizations but also in-hospital outcomes. Despite similar demographic characteristics, patients admitted with HF in two referral centers in South London in 2020 experienced worse outcomes compared with those admitted in the previous year (12). Hospitalization for HF in 2020 was independently associated with increased mortality risk (12). Similarly, in Germany, there was a decrease by 30% in urgent HF hospitalizations during the pandemic (*p* < 0.01) with a concomitant higher in-hospital mortality compared with both same-year and previous-year control groups (13).

**Table 1:** Reduction in hospitalizations due to HF during COVID-19 pandemic period, compared with same period in the previous year or a different period in the same year (before COVID-19).

**Study (year)**	**Number of patients**	**Country**	**Study and control periods**	**Reduction in HF hospitalizations**
Bromage et al. (9)	104	England and Wales	2 March−19 April 2020 vs. control period in 2020 (pre-COVID) and the same periods in 2017–2019	−66%, *p* < 0.01
Cox et al. (10)	–	Vanderbilt University Medical Center, Nashville, Tennessee	22 March−20 April 2020 vs. same period in 2019	−62 ± 7%, *p* < 0.01
Hall et al. (11)	–	USA	Mean weekly hospitalization from January 2020 to 11 April. The significant and progressive decline described in 2020 was not observed in 2019, excluding potential confounding based on seasonal trends.	−50% (after the first case of COVID-19)
Cannatà et al. (12)	1,372	South London	7 January−14 June 2020 vs. same period in 2019	−40%, *p* < 0.001
Konig et al. (13)	13,484	Germany	13 March−21 May 2020 vs. control periods in 2020 (1 January−12 March) and 2019 (13 March−21 May)	−30%, *p* < 0.01

*COVID-19, Coronavirus Disease 2019; HF, heart failure*.

## COVID-19 and Cardiovascular Comorbidities

The clinical presentation and the course of COVID-19 is extremely variable, ranging from an asymptomatic or pauci-symptomatic illness, presenting with mild symptoms (e.g., fever, dry cough, and fatigue), to a severe disease [e.g., severe pneumonia and acute respiratory distress syndrome (ARDS)] with possibly fatal outcome (14–18). The earliest reports from China and Italy showed a high prevalence of comorbidities and their association with the severity of COVID-19 and increased mortality (19–21). In a report of 72,314 cases, the overall case-fatality rate of COVID-19 was 2.3%, with higher rates in patients with pre-existing comorbidities [10.5, 7.3, 6.3, and 6.0% in patients with cardiovascular disease (CVD), diabetes, chronic respiratory disease, and hypertension, respectively] (22). A more recent meta-analysis suggested that CVD and cardiovascular risk factors (hypertension and diabetes) were closely related to fatal outcomes in COVID-19 patients, across and independently from all ages (23).

## Impact of Pre-Existing Heart Failure On COVID-19 Clinical Course

Further studies showed that the prevalence of HF as a comorbid condition ranged from 3.3 to 21% among SARS-CoV-2-infected patients (Table 2) (19–21, 24–28). In a multicenter retrospective study from New York City area, including nearly 3,000 patients with laboratory-confirmed SARS-CoV-2 infection, the prevalence of HF was 10.1% (25). HF patients were more prone to develop myocardial injury, defined as increased troponin levels. HF history was also found to be associated with an increased risk of hospitalization and a severe clinical course in COVID-19 patients. In a prospective cohort study, among 5,279 people with laboratory confirmed SARS-CoV-2 infection, more than a half were admitted to hospital, of whom 1,904 (69.5%) were discharged alive (19). Besides age, HF was one of the strongest predictor for in-hospital admission [odds ratio (OR), 4.43; 95% confidence interval (CI), 2.59–8.04; *p* < 0.001] and critical illness (OR, 1.9; 95% CI, 1.4–2.5; *p* < 0.001) (19). A retrospective analysis conducted in Spain showed that HF was associated with higher risk of mechanical ventilation and mortality among patients hospitalized for COVID-19, regardless of left ventricle ejection fraction (LVEF) (28). Similar results were found in an Italian multicenter study, with HF resulting as an independent predictor of mortality and a risk factor for in-hospital complications, namely, acute HF, acute renal failure, and multiorgan failure (27).

**Table 2:** Prevalence, incidence, and mortality of pre-existing and acute HF in COVID-19.

**Study (year)**	**Number of patients**	**Number of patients with history of HF**	**Prevalence of HF history (%)**	**Main outcome of patients with history of HF**	**Incidence of acute HF during COVID-19 (%)**	**Outcome in acute HF patients**
Inciardi et al. (21)	99	21	21	Higher mortality in HF vs. non-HF patients (57 vs. 18%, *p* = 0.009)	–	–
Shi et al. (24)	671	22	3.3	History of HF was more prevalent in dead patients vs. survivors (21 vs. 1.5%, *p* < 0.001)	–	Acute HF was the cause of death in 19.4% of cases.
Petrilli et al. (19)	5,279	367	7	Adjusted HR for death 1.77 (95% CI, 1.43–2.2, *p* < 0.001)	–	–
Lala et al. (25)	2,736	276	10.1	HR for death 1.03 (95% CI, 0.77–1.37, *p* = 0.867)	–	–
Richardson et al. (20)	5,700	371	6.9	–	–	–
Rey et al. (26)	3,080	152	4.9	Higher mortality in HF vs. non-HF patients (48.7 vs. 19%, *p* < 0.001)	2.5	Acute HF patients had higher mortality (46.8 vs. 19.7%, *p* < 0.001
Tomasoni et al. (27)	692	90	13	Adjusted HR for death 2.25 (95% CI, 1.26–4.02, *p* = 0.006)	9.1	Acute HF patients had higher mortality (40.0 vs. 21.8%, *p* = 0.004)
Zhou et al. (17)	191	–	–	–	23	Acute HF was more prevalent in dead patients vs. survivors (52 vs. 12%, *p* < 0.0001)
Alvarez-Garcia et al. (28)	6,439	422	6.6	Adjusted OR for death 1.88 (1.27–2.78, *p* = 0.002)	0.6 (*de-novo* HF)	*De-novo* HF patients had increased risk of ICU (HR, 2.2; 95% CI, 1.2–3.8) and intubation (HR, 2.2; 95% CI, 1.2–4.3), but not mortality (HR, 1.1, 95% CI, 0.6–2.0)

*CI, confidence interval; COVID-1, Coronavirus Disease 2019; HF, heart failure; HR, hazard ratio; OR, odds ratio*.

## The Pathophysiology of COVID-19 Myocyte Injury

### Indirect Mechanisms

**Figure 1:**
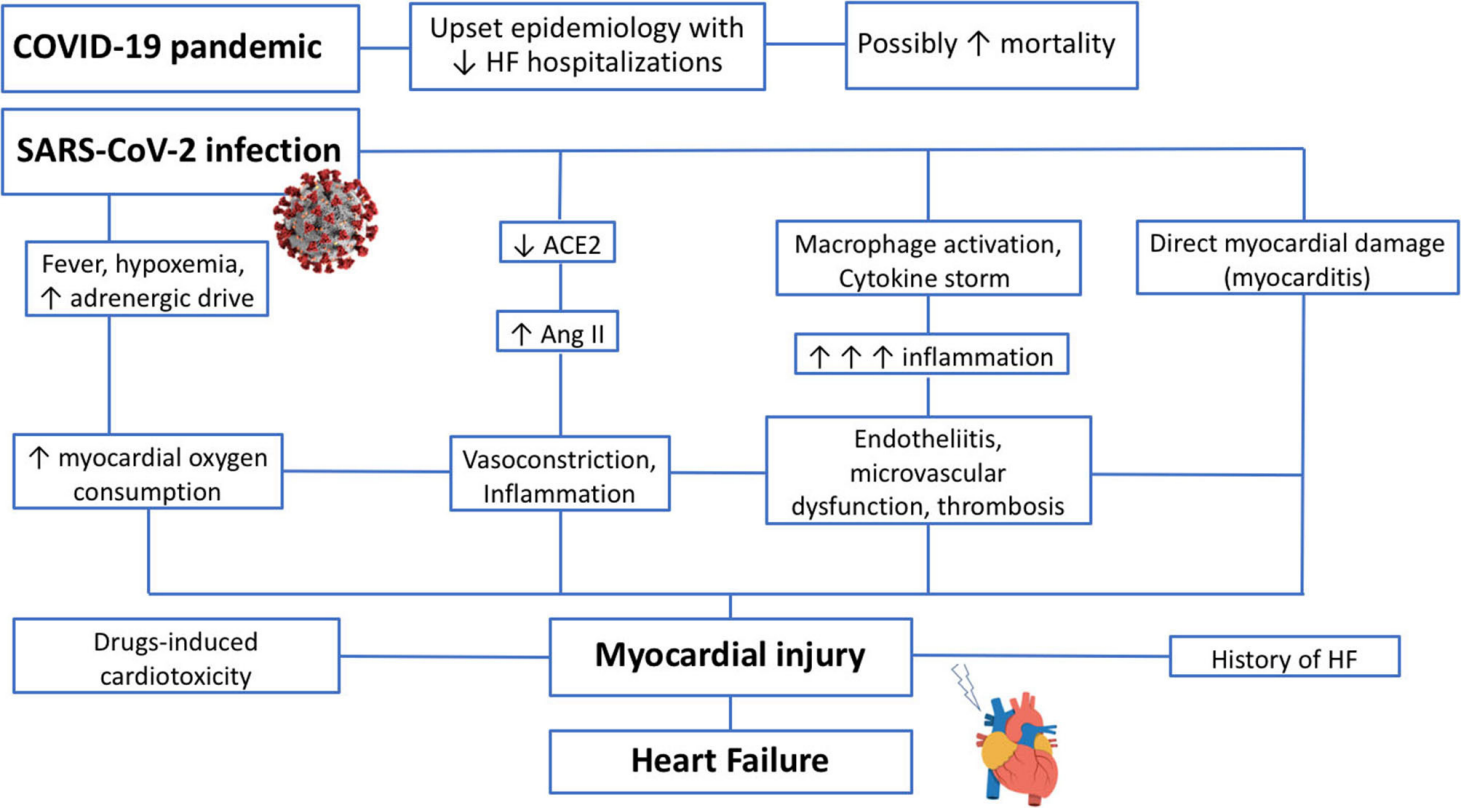
COVID-19 and Heart Failure: mechanisms of myocardial damage in COVID-19. ACE2, angiotensin-converting enzyme2; Ang II, angiotensin II; COVID-19, coronavirus disease 2019; SARS-CoV-2, severe acute respiratory syndrome coronavirus 2; HF, heart failure.

The pathogenesis of myocardial injury in COVID-19 is still not completely clear and likely involves multiple pathways. Overall, myocardial damage can be summarized distinguishing two different mechanisms of injury: the first, “indirect” or “aspecific,” common with other severe infections, and the second, “direct” or “specific,” related to the peculiar effects mediated by SARS-CoV-2 (3). The mechanisms of myocardial damage are highlighted in Figure 1.

First, COVID-19 has general deleterious effects on the cardiovascular system, which were already described in other infections (i.e., influenza and community-acquired pneumoniae). Fever and sympathetic activation cause tachycardia with a consequent increase in myocardial oxygen consumption (29–31). Moreover, prolonged bed rest and systemic inflammation favor coagulation disorders. Both venous and unusual arterial thromboembolic events were observed in COVID-19 patients (32, 33). Hypoxemia, another hallmark of COVID-19, is associated with enhanced oxidative stress with reactive oxygen species production and subsequent intracellular acidosis, mitochondrial damage, and cell death (29, 34).

A second series of indirect mechanisms are those related with the peculiar abnormal inflammatory response that COVID-19 may elicit: the presence of a proinflammatory surge, the so-called cytokine storm, may happen in a week after the infection and is thought to be central in the pathogenesis of the acute lung injury/ARDS spectrum, as it is reported in severely ill patients (35, 36). Indeed, during the acute phase of the infection, an imbalanced response of types 1 and 2 T helper cells may lead to a hyperinflammatory response (35, 36), resulting in an excessive release of cytokines: in particular, higher levels of interleukin-1β (IL-1β), interleukin-6, interferon-γ, tumor necrosis factor (TNF), macrophage inflammatory protein, and vascular endothelial growth factor (VEGF) have been described in patients affected by severe COVID-19 (16–18), and are independently associated with a severe course of the infection and eventually death (16, 37). In addition, the hyperinflammation syndrome seems to be pivotal in the development of cardiac injury, since a positive correlation has been described between the increase in inflammatory markers and myocardial damage in COVID-19 (38–41). Consistently, previous *in-vitro* studies have shown that the release of proinflammatory cytokines such as TNF and IL-1β, in other septic conditions, were responsible for myocardial cells depression (42–44), through modulation of calcium channel activity and nitric oxide production (43, 44).

Cytokine storm may be as well the cause of acute HF: the inflammatory activation and oxidative stress are similarly present in HF and may predispose, combined with COVID-19, to a more severe clinical course (45–47). Finally, the marked inflammatory response takes place also in the endothelium, as demonstrated by post-mortem histological findings showing lymphocytic endotheliitis with apoptotic bodies and viral inclusion structures in multiple organs (48, 49). Endotheliitis can lead to disseminated intravascular coagulation with small or large vessels thrombosis and infarction and significant new vessel growth through a mechanism of intussusceptive angiogenesis (49, 50).

Consequently, anti-inflammatory therapies and thromboprophylaxis have been the mainly studied drugs for COVID-19 (51–54). Dexamethasone was found to be associated with lower 28-day mortality in the controlled, open-label Randomized Evaluation of COVID-19 Therapy (RECOVERY) trial (51). Beneficial effects were limited to those patients receiving ventilatory support (either invasive or non-invasive), while neutral effects were reported among patients not requiring oxygen therapy. The efficacy of steroids was confirmed in further retrospective series and in one meta-analysis of seven randomized trials, including 1,703 patients (53).

Finally, drugs that have been used as COVID-19 therapy may cause themselves myocardial injury. At the beginning of the pandemic, many drugs were proposed in an expedited manner (55). Hydroxychloroquine was initially proposed as an effective drug for the therapy of COVID-19. It is known that hydroxychloroquine has cardiovascular toxicity, as it may cause arrhythmias and heart failure (56). A recent meta-analysis, including a total of 5,652 COVID-19 patients, showed that treatment with hydroxychloroquine or chloroquine was associated with risk of drug-induced QT prolongation and higher incidence of torsades de pointes, ventricular tachycardia, or cardiac arrest (57), while no efficacy was found in the treatment of hospitalized patients with COVID-19 for hydroxychloroquine in the RECOVERY trial (58). Similarly, azithromycin was initially recommended in patients with COVID-19, but it may increase the risk of adverse CV events (high risk of QTc prolongation, especially when administered concomitantly with hydroxychloroquine (59). Several antiviral drugs are known to cause mitochondrial dysfunction and cardiotoxicity (60, 61).

### Direct Mechanisms: the Role of ACE 2

Angiotensin-converting enzyme (ACE)2 is the key to understand the consequences of SARS-CoV-2 infection on the CV system. ACE2 is a membrane protein, that is highly expressed in different organs, including heart, lungs, gut, and kidneys. It mediates SARS-CoV-2 entry into the host cells (62). Thus, ACE2 may facilitate organ damage by direct virus entry, with different clinical implications, according to the target organ. The virus, once inside the cell, uses host's ribonucleic acid (RNA)-dependent RNA polymerase to replicate its own structural proteins and, when assembled, new virus is released from the cells; as a consequence, host cell can be damaged/destroyed in this process (5). Consistently, SARS-CoV-2 positivity in cardiac tissues could be documented in autoptic studies in consecutive patients who died as a consequence of COVID-19 (63). Supportively, in engineered heart tissue model of COVID-19 myocardial pathology, SARS-CoV-2 was found capable to directly infect cardiomyocytes through ACE2, resulting in contractile deficits, cytokine production, sarcomere disassembly, and cell death (64).

In addition, ACE2 may be not only a simple bystander in the pathophysiology of myocardial injury: indeed, besides being the receptor of SARS-CoV-2, is an enzyme involved in the renin–angiotensin–aldosterone system (RAAS). Once binding is complete, the virus attaches ACE2 throughout membrane fusion and invagination, causing a downregulation in the activity of ACE2 (65). Particularly, ACE2 cleaves angiotensin II into angiotensin 1–7, which has vasodilating and anti-inflammatory effects. ACE2 has also a weak affinity for angiotensin I and can convert it into the non-apeptide angiotensin 1–9, limiting angiotensin II synthesis by ACE, and with vasodilatatory effects through angiotensin type 2 (AT2) receptor stimulation. Thus, ACE2 can counteract the untoward effects of angiotensin II with vasodilatatory, antioxidant, and antifibrotic effects (66). Interestingly, ACE2 has also immunomodulatory properties both direct, through its interaction with macrophages, and indirect, reducing angiotensin II which stimulates inflammation (67). ACE2 downregulation by SARS-CoV-2 infection may increase angiotensin II levels, favoring AT1 receptor activity, with a subsequent vasoconstriction, fibrotic, proliferative, and proinflammatory effects (3).

## Clinical Presentations of Myocardial Injury in COVID-19

COVID-19 often affects the heart. The clinical manifestations of cardiac involvement could range from an absolute lack of symptoms in the presence of increased troponin levels, with or without ECG or imaging abnormalities, to arrhythmia and sudden cardiac death, pulmonary embolism, acute coronary syndromes, myocarditis, acute HF, and cardiogenic shock (3, 68, 69). The majority of patients with cardiac injury, as assessed by serum troponin elevation, do not have clear cardiac symptoms, whereas a minority is diagnosed with myocarditis or acute myocardial infarction. In more than a half of cases, ECG abnormalities compatible with myocardial ischemia (T-wave depression and inversion, ST segment depression, Q-waves) were described (38).

Recently, it has been shown that patients with cardiac injury have a greater prevalence of left ventricle (LV), right ventricle (RV), and pericardial abnormalities (69). Diastolic dysfunction was more frequent in patients with myocardial injury, possibly reflecting the higher prevalence of hypertension, diabetes, and chronic kidney disease among those patients, known risk factors for HF with preserved ejection fraction (HFpEF) (70). Transthoracic echocardiography (TTE) abnormalities and concomitant cardiac injury were correlated with an increased risk of death; thus, TTE evaluation might be useful to characterize the underlying cardiac substrate, for risk stratification, and to guide clinical decisions (69).

## Subclinical Acute Myocardial Injury in COVID-19

Since the first Chinese reports, a high incidence of cardiac injury, defined as the presence of elevated troponin (Tn) levels above the 99th percentile of the reference interval, was found in COVID-19 patients. The prevalence of cardiac injury ranges from 12% in unselected COVID-19 cases up to 41% in critically ill patients and patients with pre-existing cardiovascular diseases with a further rise to 75.8% in non-survivors (Table 3) (14, 17, 18, 24, 25, 38, 39, 71–82). The presence of elevated Tn levels is associated with abnormal laboratory findings (including white blood cells count, neutrophil and lymphocyte count, C-reactive protein, procalcitonin, N-terminal pro-B-type natriuretic peptide, D-dimer, transaminases, lactate dehydrogenase, total bilirubin, albumin, prothrombin time, and cytokines) (18) and a higher grade of pulmonary involvement in radiographic findings, suggesting an important multiorgan involvement (21, 38, 73). This may be a consequence of the derangement in the innate and adaptive immune response, with a cytokine storm, similar to that previously observed in SARS and Middle East Respiratory Syndrome (MERS) (3). The presence of cardiac injury in COVID-19 is associated with more severe manifestations, complications, and adverse prognosis (14, 17, 18, 24, 25, 38, 39, 71–82). Data about the association between cardiac injury and mortality are summarized in Table 3.

**Table 3:** Cardiac injury prevalence and risk of in-hospital death in different geographical settings.

**Study (date of publication)**	**Country**	**Number of patients**	**Severity**	**Patients with cardiac injury (*n* (%))**	**In-hospital deaths among cardiac injury patients (*n* (%))**	**HR (95%CI) for death in cardiac injury group**	**OR (95% CI) for death in cardiac injury group**
Huang et al. (18)	China	41	Mixed (inpatients)	5 (12%)	–	–	–
Shi et al. (38)	China	416	Mixed (inpatients)	82 (19.7%)	42 (51.2%)	3.41 (1.62–7.16)	–
Guo et al. (39)	China	187	Mixed (inpatients)	52 (27.8%)	31 (59.6%)	–	–
Zhou et al. (17)	China	191	Mixed (inpatients)	– (17%)	–	–	80 (10.3–620.4) (univariate)
Chen et al. (14)	China	274	Moderate to severe/critical	– (41%)	–	–	–
Deng et al. (71)	China	112	Mixed (inpatients)	42 (37.5%)	14 (33.3%)	8.9 (1.9–40.6) (peak troponin T)	–
Wei et al. (72)	China	101	Mixed (inpatients)	16 (15.8%)	3 (18.8%)	–	6.63 (2.24–19.65) (univariate) (progression to severe disease)
Shi et al. (24)	China	671	Severe and critical	– (15.8%)		4.56 (1.28–16.28) (multivariate)	–
Lala et al. (25)	USA	2,736	Mixed (inpatients)	Troponin I >0.03 to 0.09 ng/ml: *n* = 455 (16.6%); troponin I > 0.09 ng/dl: *n* = 530 (19.4%)	–	1.75 (1.37–2.24); 3.03 (2.42–3.80)	
Lombardi et al. (73)	Italy	614	Mixed (inpatients)	278 (45.3%)	104 (37.4%)	1.71 (1.13–2.59) (multivariate)	–
Calvo-Fernandez et al. (74)	Spain	872	Mixed (inpatients)	(34.6%)	(29.3%)	2.91 (1.21–7.04) (multivariate)	–
Fan et al. (75)	China	353	Mixed (inpatients)	79 (22.4%)	45 (57%)	1.65 (1.17–2.34) (multivariate)	–
Giustino et al. (69)	International (USA, Italy)	305	Mixed (inpatients with TTE and ECG)	190 (62.3%)	51 (26.8%)	–	6.67 (2.76–16.11) (univariate)
He et al. (76)	China	1,031	Mixed (inpatients)	215 (20.7%)	131 (60.9%)	–	2.34 (1.23–4.45) (multivariate) (among severe patients)
De Almeida et al. (77)	Brazil	183	Mixed (inpatients)	–	–	1.12 (1.03–1.47) (multivariate)	–
Manocha et al. (78)	USA	446	Mixed (inpatients)	112 (25.1%)	51 (45%)	–	4.38 (2.32–8.28) (multivariate) (30-day in-hospital mortality)
Bardaji et al. (79)	Spain	186	Mixed (inpatients)	41 (22%)	–	3.54 (1.70–7.34) (multivariate)	–
Efros et al. (80)	Israel	320	Mixed (inpatients)	91 (28.4%)	33 (36%)	4.32 (2.8–8.99) (multivariate)	–
Ali et al. (81)	Pakistan	466	Mixed (inpatients)	168 (36%)	130 (77.4%)	3.61 (0.70–1.86) (multivariate)	–
Tanboga et al. (82)	Turkey	14,855	Mixed (inpatients)	1,027 (6,9%)	–	1.89 (1.62–2.21) (multivariate) (30-day in-hospital mortality)	–

*CI, confidence interval; ECG, electrocardiogram; HF, heart failure; HR, hazard ratio; OR, odds ratio; TTE, transthoracic echocardiogram; USA, United States of America*.

Chinese reports firstly described the impact of comorbidities and underlying CVD on the development of myocardial injury and subsequent fatal outcomes (15, 39). In-hospital mortality was 7.6% for patients without underlying CVD and normal Tn levels, 13.3% for those with underlying CVD and normal Tn levels, 37.5% for those without underlying CVD but elevated Tn levels, and 69.4% for those with underlying CVD and elevated Tn (39). Moreover, the mortality rate increases with the magnitude of the troponin elevation: the mortality rate is higher among patients with vs. without cardiac injury [42 (51.2%) vs. 15 (4.5%); *p* < 0.001] (39). After adjusting for multiple variables (e.g., age, pre-existing cardiovascular diseases, cerebrovascular diseases, diabetes mellitus, chronic obstructive pulmonary disease, renal failure, cancer, ARDS, creatinine levels, and NT-proBNP levels), the hazard ratio (HR) for death among patients with cardiac injury was 4.26 (95% CI, 1.92–9.49), *p* < 0.01 (39). The prognostic role of cardiac injury was confirmed in further and larger studies from different countries (including European, American, and Asiatic nations), with mortality HR ranging from 1.12 to 8.9 depending on the regression model used and OR up to 80 in a single univariate model (17, 71–82) (Table 3). Data from a recent meta-analysis including 12,262 patients from 13 studies summarized that elevated Tn is associated with increased mortality (OR, 4.75; 95% CI, 4.07–5.53; *p* < 0.001; *I*^2^ = 19.9%), with 55% sensitivity and 80% specificity (83). Therefore, Tn test offers an important prognostic tool for the stratification of the risk of mortality in patients affected by COVID-19.

## The Dilemma of Myocarditis in COVID-19

Sometimes, cardiac involvement is clinically evident, and besides elevation of serum Tn, patients complain of chest pain, palpitation, or symptoms of HF (14, 17, 84). They may develop LV or biventricular dysfunction, in the absence of obstructive epicardial coronary disease, raising the clinical concern for myocarditis. Plenty of clinical reports described cases of acute myocarditis, presenting with cardiogenic shock, as a possible manifestation of COVID-19 (85–90).

However, myocarditis diagnosis can be controversial. Most of those cases were diagnosed based on cardiac magnetic resonance (CMR) findings that may show diffuse ventricular wall thickening and edema with wall pseudohypertrophy, with or without late gadolinium enhancement. Tissue diagnosis criteria were met only in few cases with endomyocardial biopsy (EMB) showing different degrees of aspecific myocardial inflammation and limited or absent myocardial necrosis (85, 86, 88, 90–92). Tissue findings in COVID-19 related to supposed myocarditis are enlisted in Table 4 (5, 48, 85, 86, 88, 92–106).

**Table 4:** Studies reporting cardiac tissue findings in COVID-19 patients.

**Study (year)**	**Number of patients**	**Design**	**Findings**
Basso et al. (92)	21	Multicenter pathology study, post-mortem	Increased interstitial macrophage infiltration was present in 86% of the cases, whereas lymphocytic myocarditis was present in 14% of the cases
Varga et al. (48)	3	Case reports, post-mortem	Lymphocytic endotheliitis in lung, heart, kidney, and liver but no sign of lymphocytic myocarditis.
Menter et al. (93)	21	Multicenter, post-mortem	Myocardial hypertrophy (71% of cases), senile amyloidosis (29% of cases), peracute myocardial necrosis (14% cases), acute myocardial infarction (5% cases)
Lax et al. (94)	11	Single-center, prospective study, post-mortem	Myocardial hypertrophy (100%), coronary small vessel disease (54%), myocardial fibrosis (91%), focal lymphocytic infiltrate (9%)
Buja et al. (95)	3	Multicenter, post-mortem	Lymphocytic myocarditis was reported in 1 case.
Duarte-Neto et al. (96)	10	Single-center, case series, post-mortem	Cardiomyocyte hypertrophy (90%), myocardial fibrosis (90%), previous myocardial infarction (40%), interstitial oedema (90%) myocarditis (20%), and fibrin thrombi (20%)
Bradley et al. (97)	14	Multicenter, case series, post-mortem	Cardiac findings were mostly non-specific: fibrosis (100%) and myocyte hypertrophy (93%). Myocarditis was present with aggregates of lymphocytes surrounding necrotic myocytes in 7%
Rapkiewicz et al. (98)	7	Single-center, case series, post-mortem	1 case had focal acute lymphocyte-predominant inflammation in the myocardium. Otherwise, cardiac histopathological changes were limited to minimal epicardial inflammation (*n* = 1), early ischemic injury (*n* = 3), and mural fibrin thrombi (*n* = 2)
Grosse et al. (99)	14	Single-center, case series, post-mortem	Myocardial hypertrophy (92.9%), acute myocardial infarction (21.4%), focal myocardial fibrosis (42.9%), amyloidosis (7.1%), mononuclear inflammatory cells in the myocardial interstitium (100%)
Hanley et al. (100)	10	Multicenter, case series, post-mortem	Acute coronary thrombosis (10%), thrombi in the microcirculation (56%), aright atrial thrombus (11%). Pericarditis (22%); marantic endocarditis in 11%
Oprinca et al. (101)	3	Single-center, case series, post-mortem	Mild to moderate perivascular edema, vascular congestion, small number of scattered lymphocytes between the myocardial fibers
Sala et al. (86)	1	Case report with EMB	Diffuse T-lymphocytic inflammatory infiltrates with huge interstitial oedema and limited foci of necrosis. No replacement fibrosis
Tavazzi et al. (88)	1	Case report with EMB	Low-grade interstitial and endocardial inflammation, with macrophages containing virions of coronaviruses. Cardiac myocytes showed non-specific features consisting of focal myofibrillar lysis and lipid droplets.
Escher et al. (102)	104	Multicenter, EMB study	5 EMBs were positive for SARS-CoV-2 E-gene-specific sequences. Other findings were active myocarditis (13.4%), inflammatory cardiomyopathy (32.6%), borderline myocarditis (2.9 %); dilated cardiomyopathy (41.3%), and amyloidosis (9.6%)
Lindner et al. (63)	39	Cohort study, post-mortem	Viral presence within the myocardium could be documented in 41% but was not associated with an influx of inflammatory cells
Kawakami et al. (103)	15	Literature review, post-mortem	None of the cases met the criteria of myocarditis, although in 3 cases microvascular infarction was described. In 2 cases, the virus was detected by RT-PCR in the atria, but no inflammation was described.
Haslbauer et al. (104)	23	Multicenter, post-mortem	60% of cases had myocardial RT-PCR positivity by SARS-CoV-2 PCR. Significantly higher levels of capillary fibrin deposition, capillary dilatation, and parenchymal microhemorrhages (consistent with microvascular dysfunction) compared with 10 autopsies without SARS-CoV-2. Five cases presented with increased cardioinflammatory infiltrate presented but without cardiomyocyte necrosis. Only while 1 case presented with active lymphohistiocytic myocarditis.
Bearse et al. (105)	41	Single-center, consecutive cases, post-mortem	Cardiac infection by SARS-CoV-2 (assessed by RT-PCR) was present in 30/41 cases. Cardiac infection by SARS-CoV-2 is associated with more cardiac inflammation (monocytes and macrophages). Four cases met criteria for myocarditis.
Fox et al. (106)	10	Single-center, case series, post-mortem	No evidence of lymphocytic myocarditis. In the COVID-19-affected cases, diffuse number of infiltrative cells of monocytes/macrophage lineage was noticed, with upper quantiles as compared to both matched control hearts.

*EMB, endomyocardial biopsy; SARS-CoV-2, severe acute respiratory syndrome coronavirus 2; RT-PCR, reverse transcriptase-polymerase chain reaction*.

SARS-CoV-2 was shown within macrophages, but not in cardiomyocytes, in an earliest case report of clinically suspected acute myocarditis (88). Further studies documented viral invasion and necrosis of myocytes (5, 107). In a series of 104 EMB, performed in COVID-19 patients with suspected myocarditis or unexplained HF, SARS-CoV-2 was identified through RT-PCR only in five samples (102). In a multicentric post-mortem study, Basso et al. found that the most common cardiac autoptic evidence in patients dying for COVID-19 was aspecific interstitial macrophage infiltration (in 86% of cases), whereas 14% of the patients presented a multifocal lymphocytic myocarditis (92) (Table 4). A literature review, identifying 277 cardiac autopsies from 22 studies, showed that the prevalence of myocarditis in SARS-CoV-2-infected patients was 1.4% (108). The most common histopathologic findings were myocardial hypertrophy (from 70 to 100% of cases) and myocardial fibrosis (80–100% of cases). Lymphocytic infiltrate and pericarditis were present in <25% of cases, while common findings were endothelitis, macro- or microvascular thrombi, and macrophage infiltrate (86% of cases) (108). Macrophage infiltration is an aspecific inflammatory histological finding, which can be found also in normal human hearts or in patients dying from bacterial sepsis and may be due to systemic hyperinflammatory response or other underlying disease rather than COVID-19 itself (109, 110).

Although a few cases of direct virus-related myocarditis may exist, the most common cardiac findings were non-myocarditis inflammatory infiltrate and single cell ischemia, showing how multiple and complex mechanisms are responsible for myocardial injury in COVID-19 patients, as stated above (67, 111–113).

## Cardiovascular Involvement After COVID-19 Vaccination

COVID-19 vaccines are a critical tool for controlling the ongoing global pandemic. In large, randomized controlled trials, COVID-19 vaccines were found to be safe and efficacious in preventing symptomatic, laboratory-confirmed, COVID-19. However, many adverse effects, namely, CV complications, have been described. Myocarditis may be a complication after mRNA COVID-19 vaccination (i.e., Pfizer-BioNTech and Moderna). Up to June 2021, more than a thousand cases of possible myocarditis and pericarditis have been signaled to the Vaccine Adverse Event Reporting System (VAERS) in the USA^1^. The cases are rare (hundreds of millions of doses have been administrated up to now) and were reported to be more common in young and adolescent males and after the second dose of the vaccine^1^. Similarly, the European Medicines Agency (EMA) reports less than a thousand cases of myopericarditis up to June 2021^2^. The cases described in literature usually present with fever, chest pain, and dyspnea, together with changes on the electrocardiogram and cardiac magnetic resonance findings consistent with myocarditis; the symptoms usually resolved rapidly (114–120). The patients were treated with non-steroidal anti-inflammatory drugs (NSAIDs) only, but, in some cases, required intravenous immune globulin (IVIG) and corticosteroids (114–120). However, up-to-date, no causal relationship between vaccine administration and myocarditis has been established.

The Centers for Disease Control (CDC) continues to recommend COVID-19 vaccination for everyone 12 years of age and older^1^. Another possible but life-threatening complication of COVID-19 vaccination is vaccine-induced thrombotic thrombocytopenia (VITT) (also referred to as vaccine-induced prothrombotic immune thrombocytopenia or thrombosis with thrombocytopenia syndrome); VITT is characterized by thrombosis, often in unusual sites (specifically, cerebral venous sinus thrombosis or thrombosis in the portal, splanchnic, or hepatic veins), and concomitant thrombocytopenia (121). The cases reported were noticed after adenovirus-based vaccination (i.e., AstraZeneca or Johnson & Johnson/Janssen) in patients without prior exposure to heparin (122–124). The majority of patients in these cases were women younger than 50 years of age, some of whom were receiving estrogen-replacement therapy or oral contraceptives (122–125). The pathogenesis is similar to heparin-induced thrombocytopenia (HIT): the enzyme-linked immunosorbent assay test in these cases identified antibodies directed against the platelet factor 4 (PF4)-heparin complex which activate platelets, similar to HIT antibodies (121–125). Patients usually presented with a median platelet counts at diagnosis of 20,000–30,000/mm^3^ with concomitant high levels of D-dimer and low levels of fibrinogen; almost 40% of the patients died, some from ischemic brain injury or superimposed hemorrhage (122–125). In the case of cerebral venous sinus thrombosis (CVST), patients usually presented with headache and progressive lethargy. The consensus treatment is based on the administration of intravenous immunoglobulin or corticosteroids (126).

The estimated incidence of CVST is 3.6 per million people after the AstraZeneca COVID-19 vaccine and 0.9 per million people after Johnson and Johnson vaccine (which is much lower than the rate of CVST in COVID-19, estimated at 207 per million) (127). According to the EMA, the risk of death and serious outcomes of COVID-19 (including thrombosis) outweighs the risk of VITT^3^.

## Heart Failure as a Consequence of COVID-19

Acute HF was found to be a possible consequence of COVID-19, with a dramatic impact on mortality (26). During COVID-19 hospitalization, about one-third of patients with previous HF had an acute decompensation of HF (27); however, acute HF can be developed not only as a decompensation of chronic HF but also as a new-onset HF (128) (Table 2). In an Italian multicenter study, acute HF occurred in 9.1% of patients during hospitalization for COVID-19, and almost half of them were “*de-novo*” HF in patients with no HF history (27). Among 3,080 consecutive patients with confirmed COVID-19 infection hospitalized in a tertiary center in Madrid (Spain), 2.5% of patients were diagnosed with acute HF and suffered from significantly higher mortality as compared with patients without HF(46.8 vs. 19.7%; *p* < 0.001) (128). Arrhythmias during hospital admission and chronic HF were the main predictors of acute HF; however, 77.9% of acute HF did not have a previous history of HF (128).

In COVID-19 patients presenting acute HF, LV systolic function is not usually compromised; on the contrary, impairment of right ventricular (RV) systolic function and LV diastolic function can be found (129). Out of 100 patients hospitalized for COVID-19, 32% were reported to have normal echocardiography, whereas 39% presented RV dilatation and dysfunction and 16% LV diastolic dysfunction, whereas reduced LV EF was reported only in <10% (130). Similar results are described in other small series (131, 132) and in a large international cohort study (69). Accordingly, LV diastolic impairment with elevated LV filling pressures (E/e' ratio) could be observed in a quarter of patients admitted for COVID-19 (132). Consistently, patients hospitalized with COVID-19 showed high likelihood of presence of HF with preserved ejection fraction (HFpEF) as compared with patients without COVID-19 according to the score of the Heart Failure Association (HFA) of the European Society of Cardiology (ESC), and HFpEF was found associated with cardiac structural and functional alterations and myocardial injury (133). Moreover, the longitudinal function could be impaired earlier than LVEF: in a Danish prospective multicenter cohort study, no differences were found between cases and controls from the general population regarding LVEF; on the contrary, LV global longitudinal strain (GLS) was significantly reduced (134). Speckle tracking was found to be able to identify a reduced basal LV longitudinal strain in more than a half of hospitalized COVID-19 patients (135, 136). Moreover, RV systolic function [assessed by RV longitudinal strain and tricuspid annular plane systolic excursion (TAPSE)] can be impaired in COVID-19 patients (137). A more pronounced reduction of mean values of LV-GLS and RV longitudinal strain could be found in severe COVID-19 patients, and speckle tracking analysis could predict mortality even after adjusting for multiple confounders (130, 137, 138).

## Long Term Consequences of COVID-19 on the Heart

Concerning data are emerging regarding the possibility of long-term subacute myocarditis following the recovery from SARS-CoV-2 infection and the development of HF as a long-term consequence of COVID-19 inflammatory cardiomyopathy. Follow-up clinical studies are starting to report the long-term COVID-19 consequences with many people still suffering from fatigue, dyspnea, and palpitations 3–6 months after the recovery from acute infection (139–142). In this context, imaging tests taken months after recovery from COVID-19 have shown ongoing signs of damage to the heart, even in people who experienced only mild COVID-19 symptoms. A German study suggested that 2 months after SARS-CoV-2 positivity, 78% of survivors had persistent heart involvement, of which 60% presented ongoing signs of myocarditis, revealed with cardiac magnetic resonance (CMR) (142). In a study including competitive athletes, referred to the sports medicine clinic after testing positive for COVID-19, 15% of patients had CMR findings suggestive of ongoing myocarditis and 30.8% suggestive of prior myocardial injury (143). In another CMR multicenter study evaluating 148 patients during convalescence, 2 months after severe COVID-19 infection with troponin elevation, myocarditis-like injury can be encountered in almost a half of cases (144). A large CMR cohort study among 1,597 US competitive athletes from the Big Ten Universities recently affected by SARS-CoV-2, reported 37 athletes with clinical and subclinical myocarditis (145). Echocardiographic assessment of patients with recent COVID-19 may, as well, show abnormalities in terms of higher degrees of diastolic dysfunction, lower men values of LV GLS, and presence of pericardial effusion, consistent with CMR findings, up to 2 months after COVID-19 recovery (146–148).

The meaning of those imaging findings are currently unknown; however, persistent myocardial damage and fibrosis in the subacute and chronic phases after recovery suggest that COVID-19 may be an independent risk factor for the development of HF (70). The early identification of patients with cardiac abnormalities is of pivotal importance as they may benefit from cardioprotective therapy and need different follow-up strategies.

## Conclusions

COVID-19 and HF have a strong connection that go beyond pathophysiology. First of all, COVID-19 pandemic had an impact on HF hospitalization: a reduction on hospital admission for HF has been extensively described, and this may have an impact on HF mortality. Second, history of HF is a frequent comorbidity in patients hospitalized for COVID-19. It is associated with a higher mortality and more complications during the clinical course, and this association is independent from other variables related with HF and COVID-19 severity.

Third, we have shown the high prevalence of cardiac injury following COVID-19 which is often diagnosed only through biomarker measurements. However, besides subclinical myocardial damage, SARS-CoV-2 infection can cause myocarditis with a severe reduction of LVEF, or diastolic dysfunction in a larger number of patients. Finally, HF may be a short- or long-term consequence of COVID-19 inflammatory cardiomyopathy with a dramatic consequence on the prognosis.

## Author Contributions

LI contributed to the design and conception of the manuscript and wrote the first draft. SB, LS, and EP wrote sections of the manuscript. DT reviewed and edited the manuscript. MM and MA supervised and edited the manuscript. All authors contributed to the article and approved the submitted version.

## Conflict of Interest

The authors declare that the research was conducted in the absence of any commercial or financial relationships that could be construed as a potential conflict of interest.

## Publisher's Note

All claims expressed in this article are solely those of the authors and do not necessarily represent those of their affiliated organizations, or those of the publisher, the editors and the reviewers. Any product that may be evaluated in this article, or claim that may be made by its manufacturer, is not guaranteed or endorsed by the publisher.

## Footnotes

^1^https://www.cdc.gov/coronavirus/2019-ncov/vaccines/safety/myocarditis.html.

^2^https://www.ema.europa.eu/en/news/covid-19-vaccines-update-ongoing-evaluation-myocarditis-pericarditis.

^3^https://www.ema.europa.eu/en/news/covid-19-vaccine-astrazeneca-benefits-still-outweigh-risks-despite-possible-link-rare-blood-clots.
